# Forensic psychiatry and Covid-19: accelerating transformation in forensic psychiatry

**DOI:** 10.1017/ipm.2020.58

**Published:** 2020-05-21

**Authors:** H. G. Kennedy, D. Mohan, M. Davoren

**Affiliations:** 1 National Forensic Mental Health Service, Central Mental Hospital, Dundrum, Dublin, Ireland; 2 Academic Department of Psychiatry, Trinity College Dublin, Dublin, Ireland

## Abstract

Swift medically led scientifically informed responses to the Covid-19 epidemic nationally have been demonstrably superior to other, non-scientific approaches. In forensic psychiatry and across all psychiatric services, urgent and clinically led responses have underlined redundancies and confusions in the governance of mental health services and a vacuum in policy makers. For the future, a greater emphasis on services for patients with schizophrenia and other severe, enduring mental disorders must aim at reducing standardised mortality ratios, managing risk of violence and improving hard outcomes such as symptomatic remission, functional recovery and forensic recovery of autonomy. This will require more use of information technology at service level and at national level where Scandinavian-style population-based data linkage research must now become legally sanctioned and necessary. A national research and development centre for medical excellence in forensic psychiatry is urgently required and is complimentary to and different from quality management.

## Background

The aim of forensic psychiatrists is to manage the COVID-19 crisis in secure hospitals and prisons to prevent clusters where possible, to provide effective medical assessment and treatment where necessary, whilst continuing to manage the mental health and violence risk posed by the vulnerable and stigmatised patients we serve. It is essential to evaluate and research organisational and service responses to this crisis and to continue to develop research in this field to improve ‘hard’ outcomes, mortality and functional recovery (Kennedy *et al.* 2019; Department of Health, 2020; WHO, 2016). The Covid-19 pandemic may continue in waves for some time (Adam, 2020; Ferguson *et al*. 2020; Friston *et al*. 2020) and behavioural approaches to successful responses will be important at every level (Lunn *et al*. 2020).

Forensic psychiatry occupies the interface between psychiatry and the law. It is a subspecialty within psychiatry, which provides specialist treatment to the most severely mentally ill. Like other tertiary referral services aiming to reduce mortality and manage risk such as oncology or obstetrics, forensic psychiatry is a national service because it requires a critical mass and minimum level of activity to maintain both effectiveness and efficiency (Amato *et al*. 2013, 2017; Kennedy *et al.* 2019). Forensic psychiatrists provide care and treatment to mentally disordered offenders in therapeutically secure hospitals and in prisons, as well as specialised consultation and liaison to general adult services. Forensic psychiatry services therefore provide care and treatment to vulnerable, high-risk patients, mentally disordered offenders with a history of serious violence combined with severe mental illness with many co-morbidities, often highly treatment resistant (Goethals, 2018) and vulnerable to worse outcomes from Covid-19 infection (Volkow, 2020). Forensic psychiatrists provide in-reach clinics in prisons nationally; are responsible for providing opinions on fitness to stand trial, criminal responsibility and other issues to courts. All in-patients are detained under mental health legislation, in Ireland either the Mental Health Act, Criminal Law Insanity Act or as Wards of Court (Kennedy, 2007) now modified by emergency legislation (Government of Ireland, 2020). This in turn leads to a high level of scrutiny, cross-examination and review of practice to ensure that deprivations of liberty, restrictive and intrusive practices are lawful and provide care and treatment in the least restrictive manner possible.

In recent years, driven by new population needs, forensic psychiatry services have begun to develop new subspecialties within forensic psychiatry including forensic psychiatry for those with intellectual and developmental disabilities (Gulati *et al*. 2018), forensic psychiatry for child and adolescent patients (Flynn *et al*. 2012) and for older patients (Davoren *et al*. 2014). These are exciting developments. Forensic psychiatrists are currently striving to manage the COVID-19 epidemic amongst patients in secure hospitals and prisons (Council of Europe, 2020a) in addition to the public health issue, that is violence, which has been the main challenge for forensic psychiatrists for many years. The WHO has defined violence as a public health issue (Violence Prevention Network, 2011a); 1.6 million people die from violence worldwide every year while many times that experience injury and morbidity due to violence. Domestic violence is strongly associated with epidemics (Chandan *et al*. 2020). Violence spreads within communities in a manner similar to a virus (Violence Prevention Network, 2011b) and is best targeted with a public health approach, linking education, expertise and scientific evidence. The WHO has stated ‘without data there is little pressure on anyone to acknowledge or respond to the problem (of violence)’ (Krug *et al*. 2002). Although not all violence or suicide can be attributed to mental illness, population rates of homicide, other violence, suicide and forensic service usage can be modelled mathematically (Kennedy *et al*. 1999; O’Neill *et al*. 2005) and violence in mental illness tracks but exceeds violence in the general population (Wallace *et al*. 2004).

Forensic psychiatry in Ireland and Europe is at a turning point. At the beginning of 2020, the National Forensic Mental Health Service was in the advanced stages of preparing to move to the new forensic hospital in Portrane, Dublin (Kennedy, 2002; Kennedy *et al*. 2016; National Forensic Mental Health Services, 2019). This may be seen as representative of forensic psychiatry in many first world countries. One of the ways recommended to examine if a public health service is sufficiently resourced for the population served is to look for signs of service strain – particularly the rates at which the severely mentally ill present to prisons (O’Reilly *et al*. 2019). The unmet need in forensic psychiatry is a barometer for the under-resourcing of psychiatric services at every level of an inter-connected public mental health system. Following extensive epidemiological research and assessment of unmet needs (O’Neill *et al*. 2003; Lenihan *et al*. 2005; O’Neill *et al*. 2005; Duffy *et al*. 2006; Wright *et al*. 2006; Wright *et al*. 2008; Curtin *et al*. 2009), a research-based report was submitted to government recommending a need for 350 secure forensic beds (Kennedy *et al*. 2006). The Department of Health responded to this recommendation by commissioning 130 secure forensic beds, of which 20 were for the new field of intellectual and developmental disorders. A further 10 beds for adolescents and 30 acute and sub-acute low secure beds were added and are nearing completion. While the modern building is welcomed by patients, carers and staff alike, the limited bed numbers will likely pose a significant challenge. The development of the new NFMHS Portrane will take Ireland from 2 secure forensic beds/100,000 population to 3.5 per 100,000, still one of the lowest resources in Europe, just as the number of general adult psychiatric beds is also one of the lowest resources in Europe (Chow & Priebe, 2016). The mathematical modelling that led to the recommendation that hospitals should run at 80–85% occupancy for surge capacity (Harrison, 1994) has taken on a new urgency. We are now taking exceptional measures to create decant wards and cohorting capacity.

## Expertise and leadership in healthcare

Forensic psychiatrists are trained to give expert evidence and to be cross-examined on their evidence rationally and rigorously in the formal setting of a court of law (Kenny, 1984). The difference between a contributory expert, an interactive expert and an expert by experience is relevant. Contributory experts (‘tier 4’ expertise, capable of innovating, teaching and training based on the experience of practicing and researching at the most challenging levels) are entirely different from interactive experts, those who acquire the vocabulary of expertise without any of the practical knowledge or experience – typically managers, lawyers and journalists. Interactive experts are valuable helpers for contributory experts, often as interpreters and communicators. But their ‘expertise’ atrophies almost at once when they lose daily contact with contributory experts (Collins & Evans, 2007). The management structures of mental health services are particularly prone to errors arising from misunderstanding the nature of expertise, failing to implement medically led plans for sufficient capacity, for tiered services and excellence (Department of Health, 1968). During a period of demedicalised leadership of psychiatric services, standardised mortality ratios for severe mental illness have been worsening in many countries (Crump *et al*. 2013; Lomholt *et al*. 2019) except for a few medical interventions (Onwordi & Howes, 2018) and falling far behind the improving survival rates and successes of medically led cancer programmes (National Cancer Control Programme, 2014; National Institutes of Health National Cancer Institute, 2020) and other areas of medicine (Hauser, 2020). Medically led governance leads to better hospital improvement and patient outcomes (Ham, 2013; Veronesi *et al*. 2013).

## Health Policy: centres of excellence

Countries such as Ireland which have relatively low Covid-19 death rates are generally those with leaders who have quickly acted on expert advice – complex, imperfect, tolerant of diversity and capable of compromise. Ireland has done very well in this crisis, so far. This contrasts with populist leaderships who had ‘enough of experts; and promised simple solutions to complex questions, perfect solutions, winner take all deals and an inability to make pragmatic compromises, with an intolerance of other points of view. Ireland has performed well by following expert advice concerning how to manage the Covid-19 epidemic so far. For the next steps in the COVID-19 crisis, as well as any future pandemics, success will depend on accepting the necessity of expertise, recognising scientific facts about epidemiology, and distinguishing experts from pundits (Collins & Evans, 2007).

The Irish Government appointed a stellar list of academic and professional leaders to produce the Fitzgerald report to examine the needs for acute hospital beds (Department of Health, 1968). This identified a dichotomy between the voluntary hospitals, located in cities and closely affiliated with Universities and medical schools, and the public hospitals mostly in rural towns. Over the years, the gradual recognition of ‘tiered’ specialist services (in Ireland referred to as ‘model’ services) has led to some rationalisation, particularly since the national cancer strategies and programme (National Cancer Control Programme, 2014), chaired by an overseas medical expert in the subject. These do not measure success by virtue signalling to journalists, when international standards and hard outcomes such as life expectancy are allowed to speak for themselves.

The teaching hospitals benefit from their boards, ensuring governance that is medically responsible, patient centred and able to maintain an arms-length protection from cyclical austerity policies and the consequences of confusion about the nature of expertise. These teaching hospitals have world class research-driven programmes for translational medicine with traditions of excellence and patient-centred values that are still intact. Psychiatry, however, has been almost entirely subsumed by the directly managed public sector and this sector (Department of Health, 1968) has no independent university teaching hospitals, no national research institute or research budgets, no programmes for clinical and translational research or evaluation of service development. Small, low-volume services cannot maintain safety standards and are at risk of increased mortality and morbidity, for example in surgery and obstetrics. Forensic psychiatry is unlikely to be different (Kennedy *et al*. 2019). The directly managed public sector has no tradition of excellence (Department of Health, 1968) and has only recently adopted quality initiatives. Excellence is quite different from quality. Excellence is the process of research and development that is the only means of achieving constant improvements in outcomes. It may be that the lack of ‘excellence’ programmes in psychiatry is the reason why hard outcomes – mortality (Lomholt *et al*. 2019; Uhrskov Sørensen *et al*. 2020), functional improvement, symptomatic remission – have been static or worsening for decades (Kennedy *et al*. 2019). Unsurprisingly, recruitment to psychiatry in Ireland has been in crisis for a decade. In spite of this, the virtuous circle of medical research in the clinic, development, teaching and training (Hauser, 2020) is alive and vigorous in forensic psychiatry. This underlines the potential that could be achieved if the teaching hospital system, with its independent governance body of medical experts (Veronesi *et al*. 2013) and stakeholder agencies such as departments of justice and of children, universities and criminal justice agencies were to be adopted in forensic and other national third and fourth tier psychiatry services.

## Covid-19 and emergency responses in forensic psychiatry

How has the Covid-19 crisis tested our services and our preparedness? The spread of the Covid-19 pandemic to Ireland impacted everyone including patients detained in confined places such as prisons and secure psychiatric wards. The onset of the national emergency was sharp, as was governmental and public recognition. During the initial weeks, fire fighting became the order of the day. Clinicians had to rapidly reassess how to practice safely and effectively from day to day. The switch to remote working by phone and video improved efficiencies and generated new risks. There have been many outstanding examples of nimble and adaptable behaviour. Telemedicine has been implemented almost everywhere including the courts. Meetings are leaner, more driven by the agenda and the need to reach practical decisions quickly. Triage has become rigorous and effective.

### General and acute medical services

The chronic shortage of acute and intensive care beds in general medicine was solved in Ireland by taking the large number of private hospital beds into the public system, a political act that exposed our collective tolerance of ‘normal’ trolley queues. This has not yet benefitted forensic psychiatry where severely mentally ill people in prison have prolonged waiting times for admission to hospital. Triage was already invented and rigorously applied by necessity (Flynn *et al*. 2011*a*, 2011*b*; O’Neill *et al*. 2016) and routine outcome measurement has developed ahead of other areas of psychiatry (Davoren *et al*. 2012, 2013, 2015; Eckert *et al*. 2017). In the medium to longer term, however, the transfer of private capacity into the public system raises questions and concerns. At present in Ireland the balance between public and private practice is the subject of much critique particularly from the point of view of productivity.

### Innovations in court proceedings in light of the COVID-19 crisis

In forensic psychiatry, court appearances by patients transferred overnight to telepresence (Day & Cleary, 2020). This has been hugely beneficial. Up to now patients awaiting trial were sent from forensic hospital to courts in person, even for minor procedural matters. This exposed patients to many risks and stresses, handcuffed in secure vehicles, crowded into holding cells, offered illicit substances. Large numbers of staff are also required at a time when many are self-isolating. Video-linked court appearances also reduce the risk of escape or abscond, further minimising the use of restrictive practices such as handcuffs. This positive development should remain in place long after the current crisis resolves.

### Psychiatry in prisons during the COVID-19 crisis

Forensic psychiatrists have long since established that the busiest acute psychiatric units are remand prisons (Lenihan *et al*. 2005; Wright *et al*. 2008; Curtin *et al*. 2009; Flynn *et al*. 2012; O’Neill *et al*. 2016). In these settings approximately 7.5% of prisoners will be suffering from psychosis at any given time. Hundreds of prisoners are admitted and discharged to these prisons every week in Ireland. The Council of Europe’s Committee for the Prevention of Torture (CPT) expressed concern about the growing number of mentally disordered offenders in the Irish Prison System (Council of Europe, 2020b). Addressing their psychiatric needs by screening for mental illnesses and other needs, triaging for admissions, assessing mental state and offering treatment and follow-up is a huge task, successfully sustained (O’Neill *et al*. 2016). COVID-19 is a major challenge in such a setting and both prisoners and health care professionals are in a perilous state of risk at the time of writing (Kinnear *et al*. 2020; NHS England and HMP Prison and Probation Service, 2020; WHO, 2020). The prison landings are ‘normally’ overcrowded with acutely psychotic prisoners in double occupancy cells waiting for psychiatric admissions that cannot happen for want of beds. Of 4,000 prisoners in Ireland, there are ‘normally’ between 250 and 300 prisoners on the caseload of forensic psychiatry in-reach teams, with about 30 at any time waiting for transfer to local approved centres or to the (forensic) Central Mental Hospital. The needs of these unwell men and women must be met, by either the community general adult services (for those with very minor offences) or the National Forensic Mental Health Service (for those with serious offences). Both services are seriously under-provided with admission beds when compared normatively to other European countries (Chow & Priebe, 2016). Prison in-reach psychiatric teams in Ireland have suffered Covid-19 infections and have had to isolate as close contacts.

To meet the needs of this vulnerable prisoner group, the National Forensic Mental Health Service and Irish Prison Service worked together to develop a system of telemedicine to provide video-link mental health clinics for the prisons service, to support prisoners and the prison primary healthcare teams. Triage is completed by forensic psychiatry nurses and psychiatrists in cooperation with IPS nurses. When a prisoner requires an in-person review, this is now conducted in a closed, screened visitor area of the prison to prevent transmission of Covid-19 from either the hospital team to the prison or vice versa. These arrangements are safe, efficient, prompt and will likely continue.

Older prisoners are a uniquely vulnerable group. Older prisoners have a health age similar to elders in the community that are 10 years their senior (Davoren *et al*. 2014), with multiple comorbidities of physical health and mental health and high risk of poor outcomes from COVID-19. Geriatricians (medicine for the elderly) do not visit prisons routinely, so forensic psychiatrists are often called on by prison primary care services where there is a psychiatric component to an older prisoner’s presentation.

The National Forensic Mental Health Service is working very closely with colleagues in the Irish Prison Service to deliver the best possible service, whilst acknowledging the challenge of providing safe and effective care to such a vulnerable unwell group at this time. It has never been more pressing to provide humane drug-free prisons and enough acute, sub-acute, medium-term and long-term secure forensic hospital places, with decant wards and surge capacity (McManus *et al*. 2004; O’Reilly *et al*. 2019), where effective treatment and rehabilitation is delivered under medical supervision.

### Covid-19 and enforced change in working practices in secure forensic hospital settings

The Covid-19 emergency has led to rapid changes in working practices in psychiatry (European Centre for Disease Control and Prevention, 2020) and forensic psychiatry (NHS England/Improvement 2020; Royal College of Psychiatrists 2020; Simpson *et al*. 2020) including training, hand hygiene, disinfection, physical distancing, screening staff twice a day, ending visitor access and leave for patients, cohorting, an isolation ward, telemedicine, quarantining of admissions (Simpson *et al*. 2020). In mental health services, the idea that patients should be treated by multi-disciplinary teams has never been subjected to any sort of scientific test of effectiveness, indeed it is difficult to say what would be evidence of effectiveness. The model of working in medicine, surgery, obstetrics and paediatrics is notably different, with the responsible treating consultant and nurses managing assessment, treatment and rehabilitation with referrals to specialist allied health professionals on a task-by-task basis. Hard outcomes might include earlier symptom remission, functional recovery and reduced mortality (Kennedy *et al*. 2018, 2019; Kennedy *et al*. 2018). Time taken for moves from secure hospitals to the community is easily measured in forensic psychiatry. Soft or subjective outcomes might include quality of life, ward atmosphere or self-reported recovery (Kennedy *et al*. 2019). There is evidence that psychology contributes to psychological improvement of function and reduction of risk (Richter *et al*. 2018; O’Reilly *et al*. 2019), occupational therapy contributes to quality of life (O’Flynn *et al*. 2018). But there is no evidence that multi-disciplinary team working of itself contributes anything extra to any outcome, hard or soft. The unintended consequences of MDT working combined with non-clinical governance are delays, administrative burden, role confusion, separation of responsibility from decision making (O’Shea, 2009), and most of all the inefficient diversion of professional therapy time from patients into team meetings, administrative and regulatory functions, risk aversiveness and other forms of waste (Meehl, 1973; Barrett *et al*. 2009; Tyrer *et al*. 2009). The Covid-19 crisis has stripped away many layers. Day-long ward rounds have been replaced by shorter focused work-rounds.

### Health and well-being for healthcare workers

This is an idea that has at last found its moment. As with every other employer, the National Forensic Mental Health service must pay greater attention to promoting employee safety. Public health information is made available to increase awareness of the new risks associated with Covid-19. Training in the donning and removal of PPE and hand washing is the new normal. Support must also be made available for impacted employees. For the future, there should be a return to investing in career development – every trainee should be able to register for a higher degree relevant to their practice. This would be a real stimulus to the knowledge culture and economy of medicine and health professions.

### Forensic psychiatry and Covid-19 driven change

There will be no return to the old normality. It may take longer than expected to achieve sufficient population immunity through vaccination or exposure. There will likely be further, lesser epidemics and there may be more deadly ones. The universal recognition of medical need and medical leadership in the face of deadly illnesses needs to be kept in the forefront of policy and practice when the increased and increasing mortality of the severely mentally ill has been missed by lay policy makers until now (Crump *et al*. 2013; Lomholt *et al*. 2019; Uhrskov Sørensen *et al*. 2020). Whole population databases have been legally permitted for decades in Scandinavia, generate the best data on mental illness as an endemic and ‘slow epidemic’ (Uhrskov Sørensen *et al*. 2020) and must become the norm across Europe now.

Shared office space is already a thing of the past. Telemedicine may replace most but not all prison in-reach and outpatient clinics, and hospital practice will benefit from leaner, more focused patient reviews. Training for psychiatrists will likely revert to the model of valuing preliminary years of training in general medicine or general practice. Out-patient and hospital work will be far more efficient through the liberation of clinicians from administrative burdens and a greater task orientation towards achieving clinical goals. Formal productivity monitoring through the use of electronic clinical management systems will drive further reform of the deployment of all health professionals. Waiting lists will be managed through the deployment of senior clinicians to this key decision-making function, with re-medicalisation of assessment and neuropharmacological management.

Forensic psychiatry needs a governance structure in which medical leaders (Ham, 2013; Veronesi *et al*. 2013), Departments of Justice and Children, Irish Prison Service and the courts have a direct influence on how resources are prioritised by the health service, and how resources are allocated to meet the needs of those with mental disorders who are before the courts and in prisons. A national centre of excellence in forensic psychiatry should be twinned with the new Central Mental Hospital.

## Summary

Prior to the Covid-19 pandemic, there was tolerance for a homeless, mentally ill and substance misusing population who cycle between psychiatric and addiction services, prisons and street services (Maxmen, 2020). They represent a pool of risk to themselves and others that may now be seen differently (Baggett *et al.* 2020). There was prior awareness of seasonal excess mortality (EuroMOMO, 2020). Trolley queues in emergency departments have now vanished overnight, but waiting lists of severely mentally ill people in prison remain (Giblin *et al*. 2012; O’Neill *et al*. 2016; Gulati *et al*. 2018) with high risks of suicide for those with substance misuse (Iqtidar *et al*. 2018) and criticism by international human rights bodies (Council of Europe, 2020a). This may prompt a greater sense of social responsibility and should prompt social intervention and prevention in ways that have not happened for the late 20th century/early 21st century epidemics of psychosis and drug-related morbidity, co-morbidity and mortality.

The future can be bright for patients in forensic psychiatry services. A new hospital will replace the 1850s Dundrum buildings in 2020. New teaching and research programmes will be an intrinsic part of the new hospital (Kennedy *et al*. 2018). This will offer opportunities to develop the next generation of forensic psychiatrists by supporting junior doctors to develop skills in research, service evaluation and service development as part of their clinical practice as consultants. We envisage a modern service where evidence-based practice is core and junior doctors have the opportunity to undertake higher degrees in research as standard. The COVID-19 crisis has had the effect of bringing about accelerated and radical changes including telemedicine and other new ways of working to increase efficiency. There is a large research agenda (Holmes *et al*. 2020). Revision of governance structures (Ham, 2013; Veronesi *et al*. 2013) to recognise the medical nature of psychiatric services for the severely mentally ill and impaired also requires consolidation. Forensic psychiatry shares common goals and inter-dependencies with other psychiatry services for the same population. We have a common cause in advocating together for more effective services for our patients, oriented towards reducing inequality in life expectancy for patients with severe mental illnesses and developmental disorders, preventing violence, victimisation and imprisonment.
